# Postmarketing adverse events associated with onasemnogene abeparvovec: a real-world pharmacovigilance study

**DOI:** 10.1186/s13023-025-03715-2

**Published:** 2025-05-06

**Authors:** Tianyu Chen, Qiying Chen, Jingfang Ye, Yuzhu Wu, Ting Liu, Yuezhen Zhang

**Affiliations:** 1 Quanzhou Medical College, Quanzhou, China; 2 The Second Affiliated Hospital of Fujian Medical University, Quanzhou, China

**Keywords:** Onasemnogene abeparvovec, OpenFDA, Adverse events, Pharmacovigilance

## Abstract

**Background:**

Onasemnogene abeparvovec (OA) is an adeno-associated virus vector-based gene therapy indicated for the treatment of paediatric patients with spinal muscular atrophy(SMA) with biallelic mutations in the survival motor neuron 1 (SMN1) gene. This study focused on analysis of the postmarketing adverse events(AEs) of onasemnogene abeparvovec (OA) reported in the US Food and Drug Administration public data open project (openFDA) database to assess the safety of OA in the real world and to provide a reference for the rational use of this drug in the clinic.

**Results:**

In total, 1,959 AEs were reported with “onasemnogene abeparvovec” as the primary suspected drug. The top 5 most frequent AEs were pyrexia (461 cases), vomiting (434 cases), aspartate aminotransferase increase (284 cases), alanine aminotransferase increase (260 cases), and hepatic enzyme increase (237 cases). A total of 77 alert signals were generated, 60 of which were not included in the drug label. The top 5 signals included troponin I increase ( ROR of 895.21, 95% CI: 734.43-1091.18), troponin T increase ( ROR of 313.30, 95% CI:220.85-444.44), rhinovirus infection ( ROR of 175.80, 95% CI:130.86-236.17), troponin increase ( ROR of 143.49, 95% CI:114.96–179.10), and increased bronchial secretion ( ROR of 142.71, 95% CI:96.63-210.77). Further analysis of AEs associated with gender and age differences identified 14 high-risk signals related to gender and 10 high-risk signals related to age. Female patients should be vigilant for vomiting, thrombotic microangiopathy, increased troponin T, proteinuria, haematuria, haemolytic anaemia, urinary tract infection, generalised oedema, and atypical haemolytic uraemic syndrome. Male patients should be alert to increased hepatic enzyme, increased bronchial secretion, respiratory tract infection, pallor, and increased blood creatine phosphokinase MB. Patients under 2 years of age should be vigilant for lethargy, increased monocyte count, decreased blood creatinine, and decreased neutrophil count. Patients over 2 years of age should be alert to hypertension, haematuria, rhinovirus infection, increased blood creatine phosphokinase, headache, and malaise.

**Conclusions:**

Mining of OA alert signals using the openFDA database provides supplementary information on AEs not included in the drug label. Clinical attention should be focused on common, strong-signal, and label-unmentioned AEs to optimise medication regimens and control risks in clinical use.

**Supplementary Information:**

The online version contains supplementary material available at 10.1186/s13023-025-03715-2.

## Background

Spinal muscular atrophy (SMA) is a rare autosomal recessive neuromuscular disorder. The estimated incidence of SMA is approximately 1 in 10,000 live births, with a prevalence of 1–2 per 100,000 people [1]. SMA is caused by pathogenic mutations (homozygous deletions or mutations) in survivor motor neuron gene 1(SMN1), which encodes the survivor motor neuron (SMN) protein [2]. SMN protein deficiency results in the degeneration of a-motor neurons located in the anterior horn of the spinal cord. This degeneration leads to muscle weakness, motor difficulties, and atrophy of the limbs, trunk, and respiratory muscles [3] SMA is the leading genetic cause of death in infants under two years of age and has a severe impact on the quality of life of both patients and caregivers [4]. Currently, only three approved therapeutic drugs for SMA are available worldwide, namely, nusinersen (Biogen), risdiplam (Roche), and onasemnogene abeparvovec (Novartis) [5]. Intrathecal injections of nusinersen every 4 months are required, and repeated daily oral administrations of risdiplam are required [6]. Onasemnogene abeparvovec (OA) is an adeno-associated virus vector-based gene therapy drug. In the United States, OA is approved for treating paediatric SMA patients under 2 years of age who have biallelic mutations in the SMN1 gene [7]. OA is approved in the EU for treating patients with 5q SMA who have a biallelic mutation in the SMN1 gene and a clinical diagnosis of SMA type 1 or up to three copies of the SMN2 gene [8]. OA directly targets SMA mutant genes and delivers full-length human SMN genes to motor neurons in patients via adeno-associated viral vectors. For long-term expression of SMN proteins in cells, only one intravenous administration is required. The improved muscle function and increased mobility in children suffering from SMA is expected to increase survival rates [9]. OA is a one-time and lifelong gene replacement therapy. OA may be more cost-effective than nusinersen and risdiplam, and less treatment time is required [6]. The FDA approved OA for marketing in May 2019, making OA available in more than 50 countries and regions worldwide [10].

Prior to drug launches, a series of evaluative studies on drug efficacy and safety is performed. However, due to multiple factors, such as conduction of clinical trials under different conditions, strict trial designs, stringent inclusion and exclusion criteria, relatively small patient samples, and limited follow-up observation times, some adverse reactions cannot be clearly defined, and late and rare adverse reactions are often difficult to detect [11]. Additionally, due to the low prevalence of SMA, the safety data obtained from clinical trials are limited and may not fully reflect reality. In addition, given the severity of SMA and the urgent need for effective treatment, OA was granted Fast Track, Priority Review and EMA Conditional Marketing Authorization, which accelerated the evaluation process and shortened the review time [12]. Therefore, the alert signals of adverse drug reactions should be continuously monitored, even after a drug is marketed. On the one hand, alert signal monitoring can confirm or reject preapproval safety data, and on the other hand, monitoring alert signals may provide additional information on AEs that are not included in drug labels. This approach can be used to evaluate the safety of drugs effectively and establish a balance between benefits and risks to inform clinical decisions. Furthermore, it is necessary to investigate whether OA carries any potential risks such as cardiotoxicity, haemophagocytic lymphohistiocytosis (HLH), infections, dorsal root ganglion toxicity, and chromosomal integration tumourigenesis [13].

Spontaneous reporting systems (SRSs) are currently the predominant means of AE surveillance worldwide and the main source of AE alert signal data [14]. Risk signal mining of AE spontaneous reporting system data can uncover new AEs, and the signal strength of known AEs can be assessed through this method [15]. Traditionally, AE data mining has been performed on some open AE databases. This procedure requires the downloading of all the raw data, followed by data cleaning and subsequent processing, which, to a certain extent, limits utility. In contrast, the openFDA database can be directly searched, and AE reports can be rapidly extracted through an open application program interface (API) [16]. In this study, we accessed the openFDA database to obtain OA-related AE data to identify alert signals associated with OA.

Due to potential differences in pharmacokinetics and pharmacodynamics of drugs in patients of varying genders and ages, the occurrence of AEs related to medication use may also differ. Accurately recognising these differences in gender and age is crucial for medication safety. Consequently, this study also aims to analyse potential gender and age differences associated with AEs related to the use of OA. The goal is to provide decision support for different treatment regimens for patients of diverse genders and ages.

## Materials and methods

### Data sources

The data were obtained from the openFDA database, a platform that provides access to FDA information on medical devices, drugs, and food. Original data from AE reports were imported from the FDA Adverse Events Reporting System (FAERS). AE codes in the FAERS database were adopted from the Medical Dictionary for Drug Regulatory Activities (MedDRA) developed by the International Council for Harmonization. The original reports are characterized by structured data, a high degree of organization, and a large amount of available information [17]. 

### Methods of AE report detection

We logged on to the openFDA data platform in the following order: “API"module, “Drug Endpoints” AE interface, and “Explore the API with an interactive chart”. The qualified drug name “Onasemnogene abeparvovec” was entered in the grey input box. Then, the view selection box was selected according to the study requirements. Next, “patient.drug.drugindication.exact”, “patient.reaction.reactionmeddraversionpt”, “primarysource.reportercountry”, “primarysource.qualification”, “patient.patientsex”, “patient.patientagegroup” and “serious” options were used to obtain different filter fields on OA and corresponding data from reported AEs. The search period was from the establishment of the database to April 4, 2024. A flowchart can be found in Supplementary Fig. 1.

### Methods and standards for the detection of AE signals

AE signal detection methods each have advantages and disadvantages. No single method can completely outperform or replace other methods at present. The use of combinations of different detection methods for drug-AE signal screening in the spontaneous presentation system database may increase evaluation accuracy, reduce false-positive signals, and focus on the actual AE signal. Therefore, in this study, the reporting odds ratio (ROR) and the Bayesian confidence propagation neural network (BCPNN) were simultaneously employed to calculate the AE signals. Cases where the number of reports was ≥ 3, the lower limit of the 95% confidence interval (95% CI) of the ROR was > 1, and IC-2SD was > 0 met the criteria for AE signal generation. Signal intensity increased with increasing values of the ROR and lower limits of the 95% CI [18]. The specific algorithms and criteria are shown in Tables 1 and 2. Adjusted ROR and log_2_ ROR were employed to quantify signals of gender and age differences. If the ROR is greater than 1 (log_2_ ROR > 0), the risk of the AE in female patients is higher than in male patients, and these signals are considered high-risk for females. Conversely, if the ROR is less than 1, it suggests a higher risk in male patients, and these signals are deemed high-risk for males. Comparative analysis of count data was conducted using the chi-squared test, with a *p*-value of less than 0.05 indicating statistically significant differences. The criteria for determining age difference signals are consistent with those for gender difference signals. Detailed algorithms are presented in Supplementary Table 1.

**Table 1:** 2 × 2 contingency table of proportional imbalance method

	Number of target adverse events	Number of other adverse events	Total
Target drug	a	b	a + b
Other drugs	c	d	c + d
Total	a + c	b + d	a + b + c + d

a: number of target AEs for the target drug

b: number of other AEs for the target drug

c: number of target AEs for nontarget drugs

d: number of nontarget AEs for nontarget drugs

**Table 2:** Formulas and thresholds for the ROR, RRR and BCPNN methods

Testing method	Calculation formulas		Standard
ROR	$\:\text{R}\text{O}\text{R}=\frac{(\text{a}/\text{c})}{(\text{b}/\text{d})}=\frac{\text{a}\text{d}}{\text{b}\text{c}}$		
	$$\:\text{S}\text{E}\left(\text{l}\text{n}\text{R}\text{O}\text{R}\right)=\sqrt{\begin{array}{c}(\frac{1}{\text{a}}+\frac{1}{\text{b}}+\frac{1}{\text{c}}+\frac{1}{\text{d}})\end{array}}$$		a ≥ 3and 95%Cl (lower limit) > 1 represents the generation of a signal
	$$\:95\% {\rm{Cl}} = {{\rm{e}}^{{\rm{ln(ROR)}} \pm \:1.96}}\sqrt {\begin{array}{*{20}{c}}{(\frac{1}{{\rm{a}}} + \frac{1}{{\rm{b}}} + \frac{1}{{\rm{c}}} + \frac{1}{{\rm{d}}})}\end{array}} $$		
BCPNN	$\:\gamma\:={\gamma\:}_{11}\frac{(C+\alpha\:)(C+\beta\:)}{\left({C}_{x}+{\alpha\:}_{1}\right)\left({C}_{y}+{\beta\:}_{1}\right)}$		
	$\:C=E\left(IC\right)={\text{l}\text{o}\text{g}}_{2}\frac{\left({C}_{xy}+{\gamma\:}_{11}\right)(C+\alpha\:)(C+\beta\:)}{(C+\gamma\:)\left({C}_{x}+{\alpha\:}_{1}\right)\left({C}_{y}+{\beta\:}_{1}\right)}$		a ≥ 3 and IC-2SD > 0 represent the generation of a signal
	$$\:V\left( {IC} \right) = \frac{1}{{{{({\rm{ln}}2)}^2}}}\left\{ {\left( {\frac{{C - {C_{xy}} + \gamma \: - \gamma {\:_{11}}}}{{\left( {{C_{xy}} + \gamma {\:_{11}}} \right)(1 + C + \gamma \:)}}} \right) + \left( {\frac{{C - {C_x} + \alpha \: - \alpha {\:_1}}}{{\left( {{C_x} + \alpha {\:_1}} \right)(1 + C + \alpha \:)}}} \right) + \left( {\frac{{C - {C_y} + \beta \: - \beta {\:_1}}}{{\left( {{C_y} + \beta {\:_1}} \right)(1 + C + \beta \:)}}} \right)} \right\}$$		
	$$\:IC-2SD=E\left(IC\right)-2\sqrt{V\left(IC\right)}$$		
	$$\:{\alpha\:}_{1}={\beta\:}_{1}=1;\alpha\:=\beta\:=2;{\gamma\:}_{11}=1$$		
	$$\:C=a+b+c+d;{C}_{x}=a+b;{C}_{y}=a+c;{C}_{xy}=a$$		
RRR	${\rm{RRR = }}\,\frac{{{\rm{a}}\,{\rm{ \times }}\,\left( {{\rm{a}}\,{\rm{ + }}\,{\rm{b}}\,{\rm{ + }}\,{\rm{c}}\,{\rm{ + }}\,{\rm{d}}} \right)}}{{\left( {{\rm{a}}\,{\rm{ + }}\,{\rm{c}}} \right)\,{\rm{ \times }}\,\left( {{\rm{a}}\,{\rm{ + }}\,{\rm{b}}} \right)}}$		

## Results

### Basic information on AE reports for onasemnogene abeparvovec

A total of 17,706,745 AE reports were recorded in the openFDA database as of April 4, 2024, among which 1,959 reports involved OA as the primary suspected drug. The United States had the highest number of reports (56.61%). Consumers or non-health-care professionals reported the most AEs(43.80%). The most frequently reported concomitant medications are prednisolone, nusinersen, and famotidine. Prednisolone is mainly used to reduce the hepatotoxicity of OA, famotidine is considered to reduce the gastrointestinal reactions of OA, and nusinersen is one of the main drugs used in the treatment of SMA. Basic information on OA-related AEs is presented in Table 3.

**Table 3:** Characteristics of AE reports associated with onasemnogene abeparvovec

Characteristics	*n*(%)
Sex	
Female	893(45.58%)
Male	783(39.97%)
Unknown	283(14.45%)
Reporting person	
Consumer or non-health professional	858(43.80%)
Physician	805(41.09%)
Other health professional	263(13.43%)
Pharmacist	19(0.97%)
Unknown	14(0.71%)
Reporting countries	
USA	1109(56.61%)
Britain	69(3.52%)
Russia	68(3.47%)
Japan	65(3.32%)
Germany	50(2.55%)
Hungary	37(1.89%)
Italy	37(1.89%)
France	36(1.84%)
Brazil	34(1.74%)
Unknown	454(23.18%)
Reporting time	
2018	1(0.05%)
2019	213(10.87%)
2020	475(24.25%)
2021	423(21.59%)
2022	434(22.15%)
2023	360(18.38%)
2024	53(2.71%)
Serious Outcome	
other serious	766(47.08%)
hospitalization	641(39.40%)
death	126(7.74%)
life threatening	80(4.92%)
disability	13(0.80%)
congenital anomaly	1(0.06%)
Common combination drugs	
prednisolone	407(20.78%)
nusinersen	98(5.00%)
famotidine	75(3.83%)

### Overall distribution of AEs associated with onasemnogene abeparvovec

The top 100 AEs are to be filtered. The total number of AE reports was 5,253 (owing to the detection of multiple AEs per report, the number of AEs was significantly greater than the actual number of reports). All AEs were categorized according to the preferred terms(PTs) and classified into System Organ Classes (SOCs) using the Medical Dictionary for Regulatory Activities (MedDRA) version 27.0. A total of 15 SOCs are highlighted (sorted by AE number). The top five SOCs were: investigations (1,889 cases); gastrointestinal disorders (737 cases); general disorders and administration site conditions (705 cases); respiratory, thoracic and mediastinal disorders (521 cases); and infections and infestations (433 cases). The top most frequent 10 AEs were pyrexia (461 cases), vomiting (434 cases), aspartate aminotransferase increase (284 cases), alanine aminotransferase increase (260 cases), hepatic enzyme increase (237 cases), thrombocytopenia (170 cases), platelet count decrease (140 cases), liver function test increase (116 cases), troponin I increase (113 cases) and pneumonia (112 cases), (Table 4).

**Table 4:** Overall distribution of onasemnogene abeparvovec-related AEs

System organ class	Total number of reported cases/*n*	AE (number of reported cases)
Investigations	1889	aspartate aminotransferase increased (284); alanine aminotransferase increased (260); hepatic enzyme increased (237); platelet count decreased (140); liver function test increased (116); troponin i increased (113); transaminases increased (87); troponin increased (83); blood lactate dehydrogenase increased (71); oxygen saturation decreased (60); body temperature increased (39); troponin t increased (33); white blood cell count decreased (33); blood creatine phosphokinase increased (30); gamma-glutamyltransferase increased (30); platelet count increased (30); white blood cell count increased (29); heart rate increased (28); blood bilirubin increased (22); monocyte count increased (22); C-reactive protein increased (21); blood creatinine decreased (19); weight decreased (45); weight increased (24); haemoglobin decreased (17); blood creatinine increased (16).
Gastrointestinal disorders	737	vomiting (434); dysphagia (52); constipation (39); salivary hypersecretion (22); retching (17); abdominal distension (19); nausea (67); diarrhoea (55); gastrooesophageal reflux disease (17).
General disorders and administration site conditions	705	pyrexia (461); illness (24); crying (17); fatigue (46); drug ineffective (36); death (35); asthenia (27); malaise (24); feeling abnormal (19); general physical health deterioration (15).
Respiratory, thoracic and mediastinal disorders	521	cough (86); respiratory failure (44); respiratory distress (37); rhinorrhoea (36); respiratory disorder (34); atelectasis (33); increased bronchial secretion (26); aspiration (20); hypoxia (20); productive cough (19); tachypnoea (19); acute respiratory failure (18); pneumothorax (18); choking (15); dyspnoea (96).
Infections and infestations	433	pneumonia (112); rhinovirus infection (46); covid-19 (44); nasopharyngitis (40); respiratory syncytial virus infection (40); pneumonia aspiration (29); respiratory tract infection (27); upper respiratory tract infection (23); viral infection (19); lower respiratory tract infection (18); bronchiolitis (15); influenza (20).
Blood and lymphatic system disorders	283	thrombocytopenia(170); thrombotic microangiopathy(35); neutropenia(26); leukopenia(27); anaemia(25).
Metabolism and nutrition disorders	146	decreased appetite(95); dehydration(35); hyperkalaemia(16).
Cardiac disorders	106	tachycardia(50); bradycardia(33); cardiac arrest(23);
Renal and urinary disorders	66	proteinuria(28); haematuria(21); renal impairment(17).
Psychiatric disorders	68	irritability(53); eating disorder(15).
Nervous system disorders	75	somnolence(25); lethargy(29); tremor(21).
Musculoskeletal and connective tissue disorders	58	scoliosis(30); muscular weakness(28).
Hepatobiliary disorders	64	hepatic function abnormal(33); hypertransaminasaemia(31).
Vascular disorders	70	pallor (16); cyanosis (15); hypertension (39).
Skin and subcutaneous tissue disorders	32	rash(32).

### Alert signal mining results for onasemnogene abeparvovec

Among the top 100 AEs, 77 potential alert signals of OA were screened using the ROR and BCPNN covering all adverse reactions in the OA drug label, of which 60 were not included in the drug label (see Table 5 for details). A total of 14 SOC categories were involved, among which the number of signals for investigations was the greatest (23 signals). The top 10 signals included troponin I increase (ROR of 895.21, 95%CI: 734.43-1091.18), troponin T increase (ROR of 313.30, 95% CI:220.85-444.44), rhinovirus infection (ROR of 175.80, 95% CI:130.86-236.17), troponin increase (ROR of 143.49, 95% CI:114.96–179.10), increased bronchial secretion (ROR of 142.71, 95% CI:96.63-210.77), aspartate aminotransferase increased (ROR of 69.75, 95% CI:61.45–79.18), liver function test increase (ROR of 73.73, 95% CI:61.05–89.03), hypertransaminasaemia (ROR of 68.23, 95% CI:47.78–97.42 ), alanine aminotransferase increase (ROR of 54.28, 95% CI:47.60-61.91 ), and respiratory syncytial virus infection (ROR of 63.40, 95% CI:46.30-86.81).

**Table 5:** Mining results of the potential alert signals of onasemnogene abeparvovec

SOC	PT(*n*)	ROR(95%CI)	IC(IC-2SD)
Investigations	aspartate aminotransferase increased (284)^#^	69.75(61.45;79.18)	5.62(5.19)
	alanine aminotransferase increased (260)^#^	54.28(47.60;61.91)	5.31(4.87)
	hepatic enzyme increased (237)^#^	46.86(40.88;53.72)	5.13(4.67)
	platelet count decreased (140)^#^	15.89(13.37;18.88)	3.75(3.18)
	liver function test increased (116)^#^	73.73(61.05;89.03)	5.45(4.82)
	troponin I increased (113)^#^	895.21(734.43;1091.18)	6.65(5.98)
	transaminases increased (87)^#^	45.79(36.90;56.81)	4.89(4.16)
	troponin increased (83)^#^	143.49(114.96;179.10)	5.72(4.97)
	blood lactate dehydrogenase increased (71)^*^	49.18(38.78;62.39)	4.86(4.06 )
	oxygen saturation decreased (60)^*^	13.17(10.18;17.04)	3.44(2.58)
	body temperature increased (39)^#^	21.04(15.31;28.90)	3.82(2.76)
	troponin T increased (33)^*^	313.30(220.85;444.44)	4.98(3.81)
	white blood cell count decreased (33)^*^	3.46(2.45;4.88)	1.71(0.56)
	blood creatine phosphokinase increased (30)^*^	11.056(7.71;15.86)	3.09(1.89)
	gamma-glutamyltransferase increased (30)^*^	14.69(10.24;21.08)	3.38(2.17)
	platelet count increased (30)^*^	26.11(18.20;37.48)	3.88(2.68)
	white blood cell count increased (29)^*^	8.31(5.76;11.99)	2.77(1.55)
	heart rate increased (28)^*^	3.21(2.21;4.66)	1.61(0.37)
	blood bilirubin increased (22)^*^	8.95(5.87;13.62)	2.78(1.39)
	monocyte count increased (22)^*^	70.24(46.06;107.10)	4.18(2.79)
	c-reactive protein increased (21)^*^	6.76(4.40;10.40)	2.47(1.05)
	blood creatinine decreased (19)^*^	70.44(44.76;110.88)	4.04(2.54)
	blood creatinine increased(16)^*^	2.63(1.61;4.31)	1.26(0.55)
Respiratory, thoracic and mediastinal disorders	cough (86)^*^	3.66(2.95;4.55)	1.8(1.07)
	respiratory failure (44)^*^	6.86(5.09;9.25)	2.61(1.60)
	respiratory distress (37)^*^	15.12(10.92;20.95)	3.48(2.39)
	rhinorrhoea (36)^*^	6.58(4.73;9.15)	2.53(1.43)
	respiratory disorder (34)^*^	12.99(9.25;18.24)	3.3(2.16)
	atelectasis (33)^*^	37.76(26.74;53.31)	4.21(3.06)
	increased bronchial secretion (26)^*^	142.71(96.63;210.77)	4.56(3.26)
	aspiration (20)^*^	21.71(13.97;33.75)	3.51(2.05)
	hypoxia (20)^*^	6.60(4.25;10.26)	2.44(0.98)
	productive cough (19)^*^	4.85(3.08;7.62)	2.08(0.59)
	tachypnoea (19)^*^	16.40(10.43;25.79)	3.27(1.78)
	acute respiratory failure (18)^*^	11.16(7.01;17.76)	2.93(1.39)
	pneumothorax (18)^*^	13.03(8.19;20.74)	3.06(1.53)
	choking (15)^*^	8.89(5.35;14.79)	2.65(0.98)
Infections and infestations	pneumonia (112)^*^	4.18(3.46;5.06)	1.97(1.33)
	rhinovirus infection (46)^*^	175.80(130.86;236.17)	5.24(4.25)
	covid-19 (44)^*^	2.95(2.18;3.97)	1.51(0.50)
	nasopharyngitis (40)^*^	2.54(1.86;3.47)	1.31(0.26)
	respiratory syncytial virus infection (40)^#^	63.40(46.30;86.81)	4.67(3.62)
	pneumonia aspiration (29)^*^	13.42(9.30;19.38)	3.28(2.05)
	respiratory tract infection (27)^*^	12.58(8.60;18.40)	3.19(1.92)
	upper respiratory tract infection (23)^*^	5.74(3.80;8.66)	2.31(0.94)
	viral infection (19)^*^	6.77(4.31;10.64)	2.45(0.97)
	lower respiratory tract infection (18)^*^	4.93(3.10;7.85)	2.09(0.56)
	bronchiolitis (15)^*^	47.86(28.76;79.65)	3.69(2.02)
Gastrointestinal disorders	vomiting (434)^#^	13.51(12.13;15.05	3.37(3.02)
	dysphagia (52)^*^	6.38(4.84;8.41)	2.53(1.60)
	constipation (39)^*^	2.14(1.56;2.95)	1.08(0.02)
	salivary hypersecretion (22)^*^	25.30(16.61;38.55)	3.67(2.28)
	retching (17)^*^	9.16(5.68;14.77)	2.72(1.15)
Blood and lymphatic system disorders	thrombocytopenia (170)^#^	19.05(16.27;22.31)	3.99(3.46)
	thrombotic microangiopathy (35)^#^	45.00(32.18;62.92)	4.36(3.24)
	leukopenia (27)^*^	6.22(4.25;9.10)	2.43(1.16)
Hepatobiliary disorders	hepatic function abnormal (33)^#^	10.53(7.46;14.85)	3.06(1.91)
	hypertransaminasaemia (31)^#^	68.23(47.78;97.42)	4.49(3.30)
General disorders and administration site conditions	pyrexia (461)^#^	19.54(17.58;21.71)	3.85(3.51)
	illness (24)^*^	3.74(2.50;5.60)	1.8(0.46)
	crying (17)^*^	49.93(3.10;8.06)	2.1(0.53)
Metabolism and nutrition disorders	decreased appetite (95)^*^	4.93(4.01;6.07)	2.2(1.51)
	dehydration (35)^*^	2.93(2.10;4.10)	1.5(0.38)
	hyperkalaemia (16)^*^	5.22(3.19;8.54)	2.14(0.52)
Cardiac disorders	tachycardia (50)^*^	6.46(4.88;8.561)	2.54(1.60)
	bradycardia (33)^*^	6.84(4.85;9.66)	2.57(1.42)
	cardiac arrest (23)^*^	3.07(2.04;4.64)	1.55(0.18)
Vascular disorders	pallor (16)^*^	6.45(3.94;10.56)	2.36(0.74)
	cyanosis (15)^*^	10.56(6.35;17.56)	2.8(1.14)
Renal and urinary disorders	proteinuria (28)^*^	17.75(12.22;25.79)	3.53(2.28)
	haematuria (21)^*^	6.70(4.35;10.30)	2.46(1.04)
	renal impairment(17)^#^	2.28(1.42;3.68)	1.08(0.40)
Psychiatric disorders	irritability (53)^*^	9.77(7.44;12.85)	3.06(2.15)
	eating disorder (15)^*^	7.77(4.68;12.92)	2.53(0.86)
Nervous system disorders	lethargy (29)^*^	5.61(3.89;8.10)	2.31(1.09)
Musculoskeletal and connective tissue disorders	scoliosis (30)^*^	53.84(37.50;77.31)	4.35(3.14)
	muscular weakness (28)^*^	2.78(1.92;4.04)	1.43(0.18)

^*^New signals; ^#^Expected signals

### Gender-specific signaling results

The calculations have identified 14 high-risk signals associated with gender differences, of which 9 are high-risk signals for females, including vomiting, thrombotic microangiopathy, troponin T increased, proteinuria, haematuria, haemolytic anaemia, urinary tract infection, generalised oedema, and atypical haemolytic uraemic syndrome. There are 5 high-risk signals for males, including hepatic enzyme increased, increased bronchial secretion, respiratory tract infection, pallor, and blood creatine phosphokinase MB increased (see Table 6 for details).

**Table 6:** Gender-specific signaling results

Female high-risk signals	ROR (95% CI)	Log_2_ ROR	*p*
vomiting	1.32( 1.05; 1.66 )	0.40	< 0.02
thrombotic microangiopathy	2.38( 1.10; 5.16)	1.25	< 0.05
troponin T increased	4.94( 1.69; 14.40)	2.30	< 0.005
proteinuria	2.98( 1.19; 7.46 )	1.57	< 0.02
haematuria	5.37( 1.58; 18.30)	2.43	< 0.01
haemolytic anaemia	10.69( 1.39; 82.43 )	3.42	< 0.02
urinary tract infection	7.99( 1.01; 63.22 )	3.00	< 0.05
generalised oedema^#^	/	/	< 0.001
atypical haemolytic uraemic syndrome ^#^	/	/	< 0.001
Male high-risk signals	ROR (95% CI)	Log_2_ ROR	*p*
hepatic enzyme increased	0.73( 0.55; 0.97 )	-0.46	< 0.05
increased bronchial secretion	0.29( 0.11; 0.73 )	-1.80	< 0.01
respiratory tract infection	0.39( 0.17; 0.89)	-1.38	< 0.05
pallor	0.29( 0.09; 0.90 )	-1.79	< 0.05
blood creatine phosphokinase MB increased	0.19( 0.04; 0.90 )	-2.37	< 0.05

^#^AEs occurred in females but not in males

### Age-specific signalling results

OA was approved in the United States in 2019 for the treatment of paediatric patients under the age of 2 with biallelic mutations in the SMN1 gene. However, this study found numerous instances in the openFDA database where it was used for patients over the age of 2. Therefore, we sought to statistically analyse and compare the occurrence of AEs between patients under 2 years of age and those over 2 years of age who were treated with OA. The calculations revealed 10 high-risk signals associated with age differences, of which 4 were high-risk signals for patients under 2 years of age, including lethargy, increased monocyte count, decreased blood creatinine, and decreased neutrophil count. For patients over 2 years of age, there were 6 high-risk signals, including hypertension, haematuria, rhinovirus infection, increased blood creatine phosphokinase, headache, and malaise (see Table 7 for details).

**Table 7:** Age-specific signalling results

High-risk signals at ≤ 2 years of age	ROR (95% CI)	Log_2_ROR	*p*
lethargy	3.10( 1.23; 7.81)	1.63	<0.025
monocyte count increased	4.16( 1.39; 12.43 )	2.06	<0.02
blood creatinine decreased	4.88( 1.41; 16.96 )	2.29	<0.02
neutrophil count decreased	4.21( 1.19; 14.88 )	2.08	<0.05
High-risk signals at >2 years of age	ROR (95% CI)	Log_2_ROR	*p*
hypertension	0.43( 0.21; 0.88 )	-1.23	<0.05
haematuria	0.25( 0.08; 0.76 )	-2.00	<0.02
rhinovirus infection	0.43( 0.21; 0.88 )	-1.23	<0.05
blood creatine phosphokinase increased	0.20( 0.08; 0.53 )	-2.32	< 0.001
malaise	0.25( 0.08; 0.76 )	-2.00	<0.02
headache	0.12( 0.01; 0.95 )	-3.08	<0.05

## Discussion

OpenFDA, a “search-based” API launched by the FDA on June 2, 2014, is an online public health project that allows people to search for text in databases similar to a search engine [17]. This vast data resource enables researchers and the public to access, mine, and utilize the FDA-based compilation of data for health care management [19]. The analysis of openFDA real-world data is a valuable approach for postmarketing assessment of drug safety and the identification of adverse reactions not detected in clinical trials. This study identified a total of 77 alert signals for OA through the openFDA platform. These signals include AEs already listed in the label, such as elevated aminotransferases, vomiting, thrombocytopenia, thrombotic microangiopathy, pyrexia, and increased troponin. Additionally, other AEs related to renal and urinary system disorders, gastrointestinal disorders, respiratory system disorders, cardiovascular disorders, and haematological disorders that are not included in the label were also identified (see Table 5 for details).

OA is an adeno-associated virus (AAV) vector-based gene therapy drug. The off-target effects of gene therapy include liver, haematopoietic, and cardiac toxicity. The most frequently reported adverse effects are elevated transaminase levels, vomiting, thrombocytopenia, and troponin I increase [20]. These adverse reactions are mentioned in the drug label, and we also detected related signals in this study (e.g., increases in aspartate aminotransferase, alanine aminotransferase, hepatic enzymes, liver function test, transaminase, gamma-glutamyltransferase, and troponin I; hepatic function abnormal; hypertransaminasaemia; platelet count decrease, thrombocytopenia, and vomiting). OA has black box warnings for hepatotoxicity, including acute liver failure, acute severe liver injury, and elevated liver transaminases [21, 22]. Two children in Russia and Kazakhstan died due to acute liver failure after OA treatment, as reported in 2022 by the foreign media outlets STAT and Endpoint News. A pooled analysis of five clinical trials performed by Chand et al. [23] also showed that among the patients treated with OA, 34% reported AEs related to hepatotoxicity and 90% reported alanine aminotransferase (ALT) or aspartate transaminase (AST) elevation after dosing. The present study identified nine relevant signals, including aspartate aminotransferase increased, alanine aminotransferase increased, hepatic enzyme increased, liver function test increased, transaminases increased, hepatic function abnormal, hypertransaminasaemia, gamma-glutamyltransferase increased, and blood bilirubin increased (Table 8). Liver enzymes are a group of enzyme substances present in the cells of the liver. Typically, the concentration of these enzymes is low in the blood. However, in instances of liver damage or disease, the liver cells release a greater quantity of enzymes into the bloodstream, resulting in elevated liver enzymes. In the present study, we found signals of elevated liver enzymes, which may be indicative of liver injury in the patient.The underlying causes may be associated with the OA-induced immune response or the high distribution in the liver [24]. It is also the case that some patients with SMA may have underlying liver abnormalities, which may render them more susceptible to the hepatotoxicity of the drug []. There may also be an increased risk of hepatotoxicity if the patient uses concomitant medications that may be hepatotoxic before or after treatment with OA. In this study, the most commonly prescribed concomitant drugs for OA were identified through a search of the OpenFDA database. These included prednisolone, famotidine, nusinersen, and acetaminophen.The relative reporting ratio (RRR) was employed to facilitate a comparative analysis of the results of disproportionality assessment of OA and its commonly concomitant medications with alert signals. The RRR value of a medication is typically the largest when it is most likely to be associated with the alert signal [25]. The results of the calculations indicated that nine hepatotoxicity-related signals were most likely to be associated with OA (Table 9). Therefore, tests of liver function parameters (AST, ALT, and total bilirubin) should be performed prior to OA administration, patients should be monitored for three months after OA infusion until levels return to normal, and medications that may damage the liver should be avoided. Systemic corticosteroids are required for all patients before and after OA administration to mitigate possible liver injury [21–22]. The efficacy and safety of OA in patients with hepatic impairment remain to be established, and there may be a greater risk of serious liver injury in patients with preexisting liver impairment prior to drug administration [21, 22]. Therefore, caution is advised in patients with impaired liver function. Although most AEs associated with hepatotoxicity are resolved with corticosteroid therapy, a small number of patients experience serious AEs, including liver failure and death. In addition, OA is a one-off treatment; patients cannot stop taking it. To minimize the risk of hepatotoxicity during the initial phase of gene therapy administration, hepatologists should be involved at an early stage, and better screening tools and evaluation criteria should be developed for patients to receive this therapy [26]. This study also found that increased hepatic enzyme is a high-risk signal for males. Consequently, males undergoing OA treatment may be more susceptible to liver injury, which warrants greater attention.

**Table 8:** A brief overview of the hepatotoxic signaling associated with onasemnogene abparvovec

alert signal	age				Sex		whether the event abated		concomitant medications
	0−2year	3-11year	12−17year	≥ 18year	Female	Male	Yes	No	
aspartate aminotransferase increased	110	74	42	19	137	123	81	3	prednisolone (148) famotidine (40) nusinersen (24)
alanine aminotransferase increased	104	61	26	33	127	109	72	3	prednisolone (130) famotidine (36) nusinersen (18)
hepatic enzyme increased	81	34	8	16	118	103	22	1	prednisolone (61) nusinersen (20) famotidine (19)
liver function test increased	30	27	4	14	51	43	10	0	prednisolone (37) nusinersen (5) famotidine (3)
transaminases increased	36	17	10	9	42	38	18	0	prednisolone (40) famotidine (16) nusinersen (12)
hepatic function abnormal	17	5	2	7	18	14	21	0	prednisolone (21) famotidine (11) predonine (7)
hypertransaminasaemia (31)	12	8	3	4	13	13	6	0	prednisolone (9) nusinersen (4)
gamma-glutamyltransferase increased	16	4	3	3	15	15	10	1	prednisolone (13) famotidine (9) acetaminophen (5)
blood bilirubin increased	12	6	0	1	12	8	7	1	prednisolone (10) famotidine (5) nusinersen (4)

**Table 9:** A comparative analysis of the hepatotoxic signalling ratio imbalance of onasemnogene abparvovec and its common concomitant drugs

alert signal	OA	prednisolone	famotidine	nusinersen	acetaminophen
aspartate aminotransferase increased	58.79	3.06	3.57	2.50	1.92
alanine aminotransferase increased	46.54	3.33	3.15	2.00	2.01
hepatic enzyme increased	40.78	1.74	1.25	1.57	3.43
liver function test increased	68.65	1.58	1.62	1.57	1.99
transaminases increased	43.48	2.78	1.22	2.80	1.8`1
hepatic function abnormal	10.35	4.70	3.90	0.74	0.83
hypertransaminasaemia (31)	66.64	2.48	1.07	3.16	1.56
gamma-glutamyltransferase increased	14.45	3.61	2.12	1.13	1.26
blood bilirubin increased	8.84	2.42	2.72	0.83	1.71

The potential carcinogenic risk of AAV gene therapy is an ongoing concern. AAV vectors generally do not integrate transgenes into cells after they enter them. However, in a mouse model, the vector genome integrates into multiple regulatory RNAs at the Rian locus. Hepatocellular carcinoma has been associated with this process [26]. It has also been observed that the AAV genome integrates into genes related to cell growth or transformation in dogs suffering from haemophilia A [27]. However, neither experiments in nonhuman primates nor human clinical trials have shown any evidence of carcinogenesis caused by AAV genome integration. No relevant signal was found in this study; therefore, the carcinogenic risk found in small animals does not necessarily apply to humans. However, drug-induced cancer is a significant AE. Therefore, we still need to be sufficiently vigilant about the use of this virus and continue to pay attention to the risk of cancer in patients who receive OA, and further clinical studies and long-term monitoring of patients treated with AAV vectors are needed.

In addition to the liver, another target organ susceptible to OA toxicity in mice is the heart [21]. Bitetti et al. [10] reported elevated troponin I levels in all patients treated with OA. A study by Strauss and coworkers [28] revealed that other than troponin increase, two other patients had elevated blood phosphocreatine kinase MB. Weiß et al. [29] observed abnormal echocardiographic manifestations in two patients. In the present study, cardiac-related alert signals such as troponin I increase, troponin T increase, troponin increase, blood creatine phosphokinase increased, tachycardia, bradycardia, cardiac arrest, and elevated heart rate were detected. With the exception of the increase in troponin I, which is included in the label, the remaining signals are not included in the label. Troponin I increase and troponin T increase were the strongest of all the alert signals. Cardiac troponins constitute a group of cardiac-specific proteins released into the circulation by cardiomyocytes following myocardial injury [30]. An increase in blood troponin concentrations serves as an indicator of the risk of cardiac injury. It is important to note that individuals with SMA may also experience cardiac complications, including arrhythmias and structural heart abnormalities [31]. An elevated risk of cardiac events may be associated with SMA itself. The precise causal relationship between OA and cardiotoxicity, or the increased risk of SMA-related cardiac events, remains uncertain. It is therefore recommended that cardiac-related markers be monitored before, during, and after the use of OA. Physicians should also employ medical judgment in considering all possibilities in the event of a cardiac event occurring after OA treatment, and should manage the patient accordingly to prevent a more serious presentation [30]. If needed, consultation with a cardiologist should be considered. This study also identified increased troponin T as a high-risk signal for females. Therefore, it is recommended that female patients undergoing treatment with Onasemnogene Abeparvovec (OA) should be closely monitored.

The research groups of Chand [32], Prabhu [33], and Dsilva [34] reported the AEs of thrombotic microangiopathy (TMA) in children after OA infusion, manifesting as haemolytic anaemia and thrombocytopenia with renal failure. The latter is characterized by increased serum creatinine, oliguria, hypertension, proteinuria, and oedema [35]. In this study, the corresponding alert signals (thrombotic microangiopathy, thrombocytopenia, blood creatinine increase, proteinuria, haematuria, and renal impairment) were detected. This study also indicates that thrombotic microangiopathy is a high-risk signal for females. In view of these collective findings, it is advisable to closely monitor SMA patients undergoing treatment with OA for the early detection of TMA, with particular attention given to female patients. As thrombocytopenia is a key feature of TMA, platelet counts should be regularly evaluated. In cases where TMA is clinically suspected, a paediatric nephrologist, paediatric haematologist, or paediatric intensivist should be consulted immediately for further analyses, including tests for haemoglobin levels, hypertension, haemolysis, excessive bruising, kidney insufficiency, and seizures in addition to platelet count measurements, to facilitate effective and prompt treatment [32]. Early identification of TMA is necessary since therapeutic interventions such as plasma exchange, dialysis or pharmacotherapy may be required to avoid progressive nephropathy or other serious complications in these patients, which can reduce associated morbidity and mortality [29]. Therefore, OA treatment for SMA patients should be conducted in centres with expertise in neuropaediatrics and paediatric nephrology [34].

There are numerous medications in clinical practice that may cause renal impairment. Although the label for OA does not include renal impairment, this study detected alert signals for renal and urinary system disorders. These may be associated with the occurrence of TMA following treatment with OA, or they may be related to systemic inflammatory responses, immunological reactions, or other complications induced by the drug. The study also indicated that proteinuria, haematuria, urinary tract infection, generalised oedema, and atypical haemolytic uraemic syndrome are high-risk signals for females. Haematuria is a high-risk signal for patients over two years of age. Therefore, it is recommended that renal function monitoring be conducted in patients treated with OA, especially in females and patients over two years of age. In addition, the efficacy and safety of OA in patients with renal impairment have not been proven, therefore, the risks and benefits should be carefully considered before using OA in such patients.

Galletta et al. [36] reported the case of a 3-year-old boy with SMA type I who developed haemophagocytic lymphohistiocytosis (HLH), a rare and life-threatening immune syndrome characterized by excessive immune activation, following gene replacement therapy with OA infusion, as well as uncontrolled, self-sustained activation of cytotoxic lymphocytes and macrophages, leading to excessive production of pro-autoimmune cytokines and ultimately, tissue damage and multiorgan dysfunction. This is the first HLH case described in the literature after gene therapy for OA. No corresponding signal was identified in this study. However, one case of HLH was identified in the openFDA database. Therefore, HLH is likely to be a rare AE of OA, which should be observed in the clinical application of OA, so that it can be treated in time to prevent serious consequences. AAV vectors of OA may trigger excessive inflammatory responses according to Galletta et al. This study mined alert signals for inflammatory indicators (e.g., C-reactive protein increased, white blood cell count increased, monocyte count increased, platelet count increased) and alert signals for infections (e.g.,pneumonia, rhinovirus infection, nasopharyngitis, respiratory syncytial virus infection, pneumonia aspiration, upper respiratory tract infection, respiratory tract infection, viral infection, and lower respiratory tract infection), which were not mentioned on the label. However, considering that the objective of OA gene therapy is to directly target the genetic root cause of SMA, rather than exerting its effects through the activation or suppression of the immune system, OA itself may not directly increase the risk of infection. Nevertheless, in certain circumstances, immunosuppressants such as corticosteroids may be used to mitigate an immune response to the AAV vector, which is also the most commonly reported concomitant medication in our study. These drugs may temporarily reduce the patient’s immune response, potentially increasing the risk of infection. Additionally, SMA patients may be more susceptible to respiratory infections due to muscle weakness, prolonged periods of bed rest, and respiratory issues. Furthermore, dysphagia in SMA patients may increase the risk of aspiration pneumonia. Also, intravenous injections or other therapeutic procedures may bring a transient risk of infection, especially in a healthcare setting. Therefore, in summary, while OA itself does not directly increase the risk of infection, the therapeutic processes associated with its use, the underlying health conditions of the patient, and potential immunosuppressive treatments may all be related to the risk of infection. Immune reactions and coinfections may increase the risk of serious systemic immune reactions after OA infusion, leading to more serious complications [21]. It is therefore recommended that patients should be closely monitored for signs of infection to facilitate timely intervention. The use of OA should be delayed in patients with infectious diseases until the infection resolves or is controlled. Additionally, rhinovirus infection is a strong signal and a high-risk signal for patients over two years of age. This finding warrants further attention.

In terms of adverse respiratory reactions, in addition to the above infection signals, 14 related novel alert signals were uncovered in this study, including cough, respiratory failure, respiratory distress, atelectasis, respiratory disorder, rhinorrhoea, increased bronchial secretion, tachypnoea, aspiration, productive cough, acute respiratory failure, hypoxia, pneumothorax, and choking, with a total of 425 recorded AEs. According to the STR1VE study, bronchiolitis, pneumonia, respiratory distress, and respiratory syncytial virus bronchiolitis were the most commonly reported serious AEs. However, these events were not considered to be related to OA [37]. Some of the alert signals mentioned in this study may also not be related to OA. Instead, these signals may be related to clinical manifestations or complications present in SMA patients. Therefore, whether OA can cause the above AEs needs to be further investigated.

With regard to AEs of the gastrointestinal system, vomiting is one of the most commonly reported AEs in the literature, with an incidence of 28.5% [38]. The results of this study indicate that vomiting is the second most common AE, accounting for 22.15% (434 out of 1,959) of all OA-related AEs. Vomiting is also identified as a high-risk signal for females. The exact aetiology of vomiting is unknown. Viral infection and prednisolone-induced acid reflux may be causes of vomiting. It is therefore recommended that attention should be paid to observing patients for signs of vomiting and dehydration, particularly in female patients, with appropriate management plans being formulated. Famotidine can be used for gastric protection and ondansetron can be used for vomiting as needed. It is recommended that prednisolone be redosed if a patient vomits within 30 min of starting prednisolone therapy [39]. An acute viral response, such as pyrexia, is also possible in patients receiving OA. The findings of this study indicate that pyrexia is the most common AE associated with OA, accounting for 25.05% (461 out of 1,959) of all OA-related AEs. It is therefore recommended that patients should be monitored for signs of pyrexia, and age-appropriate antipyretics may be used as necessary to control pyrexia. In patients with a normal platelet count, ibuprofen should be used. When thrombocytopenia is a concern or the patient is younger than 6 months, acetaminophen is preferred [39].

Microscopic changes in the dorsal root ganglion (DRG) after the intrathecal administration of OA have been reported in nonhuman primate (NHP) trials [40]. The use of OA in a spinal muscular atrophy phase III SPR1NT trial led to two AESIs in one child (reflex loss), which could be related to dorsal root ganglionopathy. However, the child showed no other clinical signs of this condition, suggesting that this AESI may represent a complication of the underlying SMA [41]. No other AEs or findings suggestive of ganglioneuropathy have been reported to date. Moreover, the corresponding signal was not detected in the present study. Further research is needed to determine whether OA causes dorsal root ganglionopathy.

In this study, approximately half of the AEs were reported by consumers or non-health-care professionals. The reason may be that consumers have a high awareness of the monitoring and reporting of AEs, as well as a convenient reporting method suitable for ordinary consumers. More than half of the reports came from the United States, with fewer reports from Asia, largely because openFDA data originate from the United States and Asian countries have their own large pharmacovigilance databases. According to the FDA’s definition of Serious Adverse Events (SAEs), an AE is classified as a serious AE if it results in any of the following outcomes: death, hospitalisation, disability, or permanent damage, poses a significant risk to life, results in a congenital anomaly or birth defect, or requires medical or surgical intervention to prevent permanent damage [42]. A total of 1627 SAEs were reported among the OA-related AEs, accounting for 83.05%. The highest number of reports was other serious AEs (766 cases47.08%), followed by hospitalization (641 cases, 39.40%). This finding suggests that health care professionals and family members should pay close attention to postdose reactions during clinical use, and timely follow-up measures are essential to prevent major injuries.

The major limitations of this study are as follows: Orphan drug status and limited usage: OA is an orphan drug used for treating a small patient population with rare diseases. Its usage is relatively limited, leading to a lower number of reported AEs, with a total of 1,959 AE reports to date. This limitation affects clinical decision-making by potentially underestimating the true incidence of AEs associated with OA. Database limitations: While there are several representative international spontaneous reporting systems for pharmacovigilance safety databases, including the WHO’s VigiBase, the European Medicines Agency’s EudraVigilance, and other Asian pharmacovigilance databases, our study only searched the OpenFDA database. This limitation may prevent us from capturing the full extent of AEs associated with OA and precludes a comparison of racial differences in the incidence of AEs between Europe, America, and Asia. The impact on clinical decision-making is that healthcare providers may not have a complete picture of the safety profile of OA across different populations. Statistical associations vs. causality: Both the ROR and BCPNN were used to explore alert signals for OA post-release. However, the signals generated were based solely on statistical associations and do not inevitably indicate a causal link between OA and AEs [43]. This limitation is crucial for clinical decision-making as it means that the observed associations require further clinical validation to establish a causal relationship. Reporting bias in OpenFDA: As a spontaneous reporting system, the OpenFDA database is susceptible to underreporting, missing data, and variable reporting quality, which can introduce potential bias [44]. This may affect the reliability of our findings and, consequently, clinical decision-making, as the data may not accurately represent the true safety profile of OA in the real world. To address these issues, we have taken the following steps: Supplementary database analysis: We plan to include data from other databases like VigiBase and EudraVigilance in future analyses to provide a more comprehensive view of the global safety profile of OA. Clinical validation: We recommend further clinical studies to validate the biological relevance of the signals identified through our pharmacovigilance analysis. Active monitoring: Based on the signals detected in this study, we propose an active monitoring approach to validate specific signals and to better understand the relationship between OA and AEs in real-world settings.

## Conclusions

In summary, this study analysed postmarketing AEs of OA using two statistics, the ROR and BCPNN, and mined the safety information for OA from the openFDA pharmacovigilance database. The most common AEs were pyrexia, vomiting, elevated aminotransferases, thrombocytopenia, and elevated troponin, which were consistent with the label, as well as a number of AEs not listed on the label. Further prospective clinical trials are needed to determine whether the newly identified AEs are relevant to OA. It is recommended that clinical attention should be focused on common, strong-signal, and label-unmentioned AEs to optimize medication regimens, avoid risks of use, identify AEs in a timely manner and treat them correctly, and increase drug therapy efficacy.

## Electronic supplementary material

Below is the link to the electronic supplementary material.

Supplementary Material 1

Supplementary Material 2

## Data Availability

The database used in this study is publicly available in website of https://open.fda.gov/.

## Abbreviations

95%: CI 95% confidence interval
AAV: Adeno-associated virus
AEs: Adverse events
ALT: Alanine aminotransferase
API: Application program interface
AST: Aspartate transaminase
BCPNN: Bayesian confidence propagation neural network
DRG: Dorsal root ganglion
HLH: Hemophagocytic lymphohistiocytosis
MedDRA: Medical Dictionary for Drug Regulatory Activities
NHP: Non-human primate
OA: Onasemnogene abeparvovec
openFDA: The US Food and Drug Administration public data open project
ROR: Reporting Odds Ratio
SAE: Serious adverse event
SMA: Spinal muscular atrophy
SMN1: Survival motor neuron 1
SOCs: System organ classes
SRS: Spontaneous reporting systems
TMA: Thrombotic microangiopathy
UTR: Untranslated region
