# Supplementary material for: Postmarketing adverse events associated with onasemnogene abeparvovec: a real-world pharmacovigilance study
Source: Orphanet J Rare Dis. 2025 May 6;20:215. doi: 10.1186/s13023-025-03715-2 (PMC12057001; doi:10.1186/s13023-025-03715-2)
Supplement: Supplementary file 1 — Supplementary Material 1 [file 13023_2025_3715_MOESM1_ESM.doc]

openFDA

Drug API Endpoints

adverse drug event interface

Disclaimer

Community

Data

About

Home

Explore the API with an interactive chart

Gray box input: Onasemnogene abeparvovec

the view selection box was selected according to the study requirements

patient.drug.drugindication.exact

primarysource.qualification

patient.reaction.reactionmeddraversionpt

patient.patientsex

primarysource.reportercountry

patient.patientagegroup

serious
