# Supplementary material for: Postmarketing adverse events associated with onasemnogene abeparvovec: a real-world pharmacovigilance study
Source: Orphanet J Rare Dis. 2025 May 6;20:215. doi: 10.1186/s13023-025-03715-2 (PMC12057001; doi:10.1186/s13023-025-03715-2)

The adjusted ROR was defined as the following:

| **Sex** | **Number of target adverse events reported** | **Number of other adverse events reported** |
| --- | --- | --- |
| **Female** | a | b |
| **Male** | c | d |

a: the number of female patients with target ADEs.

b: the number of female patients with target drugs but not target ADEs.

c: the number of male patients with target ADEs.

d: the number of male patients with target drugs but not target ADEs.


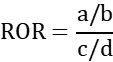

Supplement: Supplementary file 2 — Supplementary Material 2 [file 13023_2025_3715_MOESM2_ESM.doc]
